# An Overview of Interactions between Goat Milk Casein and Other Food Components: Polysaccharides, Polyphenols, and Metal Ions

**DOI:** 10.3390/foods13182903

**Published:** 2024-09-13

**Authors:** Bohan Ma, Majida Al-Wraikat, Qin Shu, Xi Yang, Yongfeng Liu

**Affiliations:** 1 College of Food Engineering and Nutritional Science, Shaanxi Normal University, Xi’an 710119, China; m18132095664@126.com (B.M.); majida@snnu.edu.cn (M.A.-W.); shuqin@snnu.edu.cn (Q.S.); 2 Department of Food Science and Technology, Tokyo University of Marine Science and Technology, 4-5-7 Konan, Minato-ku, Tokyo 108-8477, Japan

**Keywords:** casein, polysaccharides, polyphenols, metal ions, interaction, influencing factors

## Abstract

Casein is among the most abundant proteins in milk and has high nutritional value. Casein’s interactions with polysaccharides, polyphenols, and metal ions are important for regulating the functional properties and textural quality of dairy foods. To improve the functional properties of casein-based foods, a deep understanding of the interaction mechanisms and the influencing factors between casein and other food components is required. This review started by elucidating the interaction mechanism of casein with polysaccharides, polyphenols, and metal ions. Thermodynamic incompatibility and attraction are the fundamental factors in determining the interaction types between casein and polysaccharides, which leads to different phase behaviors and microstructural types in casein-based foods. Additionally, the interaction of casein with polyphenols primarily occurs through non-covalent (hydrogen bonding, hydrophobic interactions, van der Waals forces, and ionic bonding) or covalent interaction (primarily based on the oxidation of proteins or polyphenols by enzymatic or non-enzymatic (alkaline or free radical grafting) approaches). Moreover, the selectivity of casein to specific metal ions is also introduced. Factors affecting the binding of casein to the above three components, such as temperature, pH, the mixing ratio, and the fine structure of these components, are also summarized to provide a good foundation for casein-based food applications.

## 1. Introduction

Milk is one of the major foods in people’s daily lives, containing various nutrients such as protein, fat, lactose, minerals, etc. [1]. Milk contains high-quality proteins, such as casein (which constitutes approximately 80% of total milk proteins), whey protein, and serum (which cannot be obtained from other food sources) [2]. In addition, milk protein has a variety of physiological functions, such as the tendency to enhance the digestion and absorption of food in the stomach, promote the growth and development of children, and to delay osteoporosis as well as to improve the body’s immunity. In recent years, goat milk has become increasingly popular in the market due to its nutritional value, which has been reported to be higher than that of bovine milk [3]. The average mineralization content of calcium, especially in goat milk, is higher than that in bovine milk. It has found that there is 3.6 g/100 g casein in goat milk while there is only 2.9 g/100 g casein in bovine milk [4]. Goat milk contains smaller fat balls that are more conducive to promoting digestion and absorption in the human body. Additionally, the lactose content in goat milk is relatively low, making it particularly suitable for lactose-intolerant people [3,4,5,6]. In order to meet the needs of different consumer groups, the production of new multi-functional dairy products with lower-cost raw materials is required [7].

Casein is a full-priced protein present in both bovine and goat milk [8]. Casein has important biological significance because it is rich in nine essential amino acids that facilitate muscle protein synthesis [9]. In addition, the process of separating casein is usually accompanied by the distribution of minerals (especially calcium, magnesium, and zinc) in milk between casein micelles and serum components [10]. Therefore, casein can serve as a natural carrier for metal ions. The multifunctionality and enormous delivery potential of casein make it a successful candidate for widespread applications in the pharmaceutical and food industries [11,12]. During the processing of milk protein, the structure of protein may change (such as the exposure of hydrophobic groups), which would affect the functional properties of the protein. When casein is mixed with polysaccharides, polyphenols, and metal ions, various functional and nutritional benefits, such as improved stability and texture, can be obtained [13]. For example, incorporation of jujube polysaccharide has been reported to improve the stability of casein network structure [14]. In addition, the addition of polyphenols, such as delphinidin 3-O-glucoside, cyanidin 3-O-glucoside, and malvidin 3-O-glucoside, could improve the texture, color, and water-holding capacity of yogurt [15]. Moreover, fortifying milk with essential minerals can also enhance the product’s nutritional profile. Calcium, for example, is crucial for bone health, and zinc plays an important role in the immune system, especially for the growth and development of teenagers [16]. Therefore, the incorporation of different food components is considered a key approach to impart better functionalities to dairy foods. To provide a deeper understanding about how these food components (polysaccharides, polyphenols, and metals ions) affect the functionalities of dairy foods such as texture, we believe it is necessary to present an overview of the interaction mechanisms as well as the influencing factors between milk caseins and the three food components. This knowledge is considered important for developing novel casein conjugates with improved functional properties in the food industry.

## 2. Interactions with Polysaccharides

**Figure 1:**
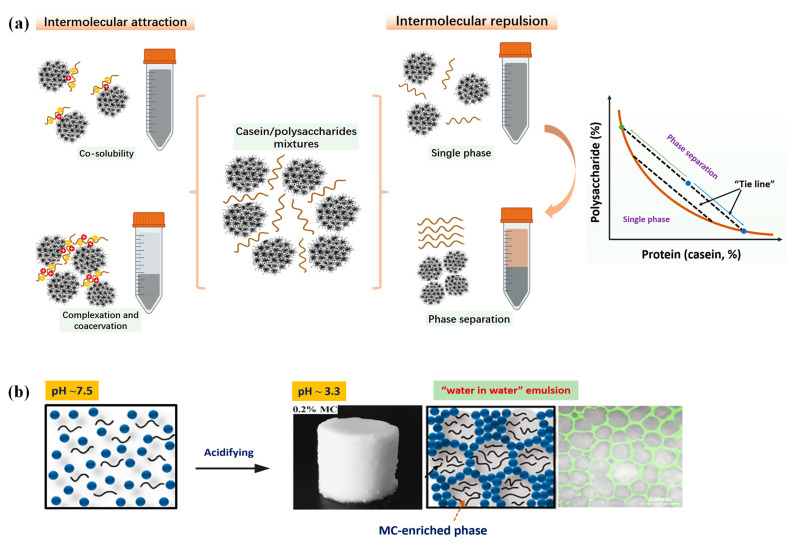
Phase behaviors and network structure of mixed milk casein and polysaccharides. (**a**) The schematic diagram of the phase transition in mixed casein/polysaccharides; (**b**) “water in water emulsion” structure of mixed casein and methylcellulose (MC) gels (8.0% casein + 0.2% MC), cited from Li et al. [19] with permission from Elsevier.

Protein-polysaccharide interactions are of great importance for biological systems and food industrial applications. Ideally, for an aqueous mixture of a polysaccharide and a protein, when all interactions are equivalent, the protein and the polysaccharide can disperse evenly in the system, meaning that each macromolecule ignores the presence of the other. However, such phenomena are only observed when polysaccharides and proteins are co-soluble either in a dilute mixture solution or when their complexes are soluble (Figure 1a). In this case, the mixture system will exhibit a macroscopic single-phase, known as co-solubility. However, the more frequent occurrence is that polysaccharides and proteins easily suffer from incompatibility due to their low mixing entropy [17]. In an aqueous mixture of polysaccharides and proteins, intermolecular attractions (e.g., electrostatic attraction) generally allow the polysaccharides and proteins to associate into soluble or insoluble complexes depending on their chemical structures and the mixing conditions [18]. Conversely, in the absence of intermolecular attractions (i.e., both polymers are negatively charged), polysaccharides and proteins will exclude each other. This repulsion yields a single homogeneous phase at low polymer concentration and a two separated liquid phases at higher polymer concentration, with each phase being enriched in one polymer, known as “water-in-water” emulsions. If the total polymer concentration is continuously increased, the two polymers will gradually separate into two isolated layers, with each layer enriching one polymer, a process called “macroscopic phase separation”.

### 2.1. Thermodynamic Aspect

So far, the thermodynamic behavior, the basic principle, as well as the theoretical models proposed to describe the protein–polysaccharide interaction have been widely studied and reviewed elsewhere [20,21,22,23,24]. In general, heterotypic interactions between two different polymers can be classified as ‘associative’ or ‘segregative’, depending on whether they are enthalpically more favorable (associative) or less favorable (segregative) than homotypic interactions between chains of the same type of polymers [24]. Association normally involves electrostatic attraction between, typically, anionic polysaccharides and proteins below their isoelectric point (pI). The driving force leading to the various phase behaviors in protein–polysaccharide mixtures is the change of the overall Gibbs free energy (∆G=∆H−T∆S, where the term “∆H” represents a positive enthalpic contribution arising from the electrostatic interactions between the biopolymers, and “−T∆S” represents a negative entropic contribution arising from the release of counterions and water molecules due to the compaction of biopolymers) [25,26]. If the total Gibbs free energy change is decreased (∆G < 0), the complexation between polysaccharides and proteins is a spontaneous process. For protein–polysaccharide complexation, the release of counterions is considered as the main driving factor because it leads to the large gain of entropy.

For the mixing systems where the intermolecular attraction is absent, there is the tendency for one type of polymer to be surrounded by the other type of polymer, which is termed “thermodynamic incompatibility”. This is a more general phenomenon and arises from the difference in the polymers’ polarity. The reason was explained by considering the pairwise interactions that can occur between two polar species: A (with dipole strength I) and B (with a higher dipole strength, I+d). Thus, the force of attraction between A and A will be proportional to $I^{2}$ and the attraction between B and B will be proportional to ${\left(I+d\right)}^{2}=I^{2}+2Id+d^{2}$, giving a total of $2I^{2}+2Id+d^{2}$ for homotypic interaction. The corresponding value for heterotypic pairing (i.e., two A–B interactions) will be $2I\times \left(I+d\right)=2I^{2}+2Id$. It is obvious that the interaction between species of the same type is more favorable by an amount that depends on $d^{2}$. Therefore, a bigger difference in the polymers’ polarity will lead to a stronger tendency for the two polymers to segregate [25]. From the viewpoint of thermodynamics, segregation into two isolate phases means a massive reduction in entropy; thus, segregation only occurs when the enthalpic advantage of segregation outweighs the entropic disadvantage.

### 2.2. Complexation between Casein and Polysaccharides

For casein, the complexation with polysaccharides has been reported to occur at a wide range of pH values, even above the pI of the protein [27]. The reason is ascribed to the existence of small “charge patches” on the surface of casein, which may carry the local positive charges and therefore bind with anionic polysaccharides [27,28]. It has also been reported that the regulation capacity of proteins may be involved, which is an intrinsic property of a protein for charge regulation. When interacting with a polyelectrolyte with opposite charges, proteins may exhibit a charge reversal from negative to positive charges, especially near the pI [29]. A typical example is the complexation of casein with κ-carrageenan, which has been reported to occur at a pH value much higher than the pI of casein (~pH 4.7–5.2) [17,30]. Since κ-carrageenan is a commonly used food polysaccharide to improve the textures of dairy products, the interaction of κ-carrageenan with milk protein (β-casein) is of interest in the food industry.

Previously, Burova et al. [31] investigated the conformational changes of ι- and κ-carrageenans as affected by β-casein at different pH values (3.00 to 7.50), ionic strengths (0.03 and 0.15 M), and molar protein/polysaccharide ratios (3–400). It was found that the complex formation of ι- and κ-carrageenans with β-casein led to the decrease in the polysaccharide ordering degree. Neither pH nor the protein/polysaccharide ratio affected the transition temperature of κ-carrageenan or ι-carrageenan in the complexes. However, the transition enthalpy of both carrageenans in complexes with β-casein decreased to zero with both decreasing pH and increasing protein/polysaccharide ratio, implying an unwinding of the polysaccharide double helix induced by β-casein [31].

Girard et al. [32] also investigated the associative phase separation process of β-lactoglobulin (β-lg)/pectin mixture solutions using small angle static light scattering under a dynamic acidification process by the addition of glucono-δ-lactone (GDL). The results suggested that acid-induced β-lg/pectin complexation followed a nucleation and growth mechanism, which involved the formation of intrapolymer and interpolymer complexes. The pectin type was found to greatly affect the structure of the complexes. The β-lg/low methoxyl pectin (LMP) complexes exhibited fractal structures, and the intrapolymer complexes possessed a more compact structure than the interpolymer complexes. In contrast, the β-lg/high methoxyl pectin (HMP) complexes are associated with rod-like structures [32]. The difference in the associated structures of the complexes was ascribed to the interaction extent between β-lg and pectin. By investigating the driving forces for forming and stabilizing the β-lg/pectin complexes, the authors further found that the complexation extent was higher for β-lg/LMP than for β-lg/HMP because LMP carried a higher charge density, which promoted its electrostatic interaction (the major driving force leading to the complexation) with the protein [33].

### 2.3. Gelation

Gelation is a process involving the transition of a polymer solution to a viscoelastic solid-like system, which occupies an important position in food application [34]. As mentioned earlier, casein is the major proteinaceous component in mammalian milk and mostly exists in solution in the form of micelles, which are unstable to heating or pH reduction, especially at low ionic strength. Therefore, slowly acidifying a casein dispersion to near its pI usually can lead to an acidic casein gel [35]. On the basis of the polysaccharide-protein interactions, investigations into the co-gelation process of mixed polysaccharides-casein systems have been carried out in the past years.

Mixing casein with neutral polysaccharides will lead to the polymer exclusion effect, therefore the aggregations of casein micelles can be greatly promoted. Previously, Li et al. [19] prepared mixed casein/methylcellulose (MC) gels by reducing the solution pH by adding GDL. At a fixed casein concentration (8.0%, *w*/*v*), it was found that with increasing amount of MC addition, the mixed gels gradually exhibited a structural conversion from a casein-dominant gel network to a “water-in-water emulsion structure”, with the casein as the continuous gelling phase and the MC as the dispersed phase (Figure 1b). With further MC addition, a bicontinuous phase structure was formed [19].

Notably, when mixing casein with anionic polysaccharides, this kind of structural transition can still be observed. By investigating the microscopic structures of composite casein/alginate gels (which were prepared by decreasing the solution pH value from ~7.0 to ~4.0), Li et al. [35] reported a similar structural transition from a casein-dominant network at low alginate concentration (0.1%) to a “water-in-water emulsion structure” (with the casein as the continuous gelling phase and alginate as the dispersed phase) at higher alginate content (0.20–0.50%). As the alginate concentration was increased to 0.75%, a bicontinuous phase structure was observed. For such a system, the authors proposed a two-step process to describe the gel formation process: (1) above the pI of the casein, the significant intermolecular electrostatic repulsion between the casein and alginate led to pronounced phase separation, which gave rise to the basic gel structures; (2) with continuous acidification, the intermolecular electrostatic association occurred between the two polymers, which led to the electrostatic association between the casein and alginate but did not change the basic phase-separated network structure [35].

Therefore, the final gel structures seem to depend on the relative ratio between the dynamics of phase separation and network development. It is considered that the structure of the mixture at the time of gelation will largely determine the final gel structure. Moreover, if two liquid phases can gel at different rates, a gel network may be formed in preference to the second network. It is believed that the formation of the first network is negligibly affected by that of the second network, but the latter can be greatly affected by the former. This is true for the mixed casein/agar double network gels as reported by Sun et al. [36]. By altering the acid gelation temperature, the authors obtained two different types of mixed gels: (1) acidifying casein-agar mixture at a temperature lower than the conformational conversion temperature of agar led to the preferential formation of an agar gel network, producing a loose casein-agar double network; (2) when acidifying the mixture at a temperature higher than the conformational transition temperature of agar, casein preferentially formed a gel network, and then agar gelled in situ in the pores of the casein network, giving a denser and more homogenous double network structure [36].

### 2.4. Other Consideration

In real mixture systems, the polysaccharide-casein interactions can lead to dynamic change of phase equilibrium as a result of several factors, which further affect the phase behaviors in the systems [24,37,38]. For example, lowering the temperature, which decreases the relative importance of entropy, promotes more complete segregation of the two polymers. Reduction in molecular weight allows a larger number of species to move independently and therefore increases the entropy weighting, favoring the mixing of the two polymers. In mixtures where one polymer is neutral and the other is charged, the addition of extraneous salt will induce phase separation [39].

### 2.5. Application

In the past few years, many researchers have demonstrated the feasibility of recovering the textures of dairy products by rational control of casein-polysaccharide interactions [40]. For example, W. Wang et al. [14] investigated the effect of jujube polysaccharide (JP) and *Lycium barbarum* polysaccharide (LBP) on the physicochemical properties of goat milk cheese, such as texture, rheological properties, and microstructure. They reported that the goat milk cheese containing 1% JP exhibited the highest water retention capacity, hardness, and rheological properties due to the formation of a more robust casein network structure. The addition of JP increased the yield of goat milk cheese, and the incorporation of LBP imparted the goat milk cheese with a lower fat content, higher moisture, and looser structure [14]. Based on the effect of JP and LBP in goat milk cheese, it is feasible to develop goat milk cheese with improved texture and good rheology, which is conducive to the development of dairy industry. Condensed milk and yogurt are increasingly favored by consumers, and these findings are conducive to the development of new flavored condensed milk and functional yogurt.

## 3. Interactions with Polyphenols

Polyphenols are a class of secondary metabolites widely existing in plants, with anti-inflammatory, antioxidant, antibacterial, and other functional properties [41,42,43]. Plant-derived phenols, including flavonoids, catechins, etc., may alleviate acute or chronic intestinal inflammation by reducing oxidative stress and pro-inflammatory states [43,44] The addition of polyphenols (e.g., kaempferol and phloretin) has been reported to increase the free radical scavenging capacity because free phenolic hydroxyl groups are introduced and more phenolic free radicals are formed, which are not energetic enough to promote lipid oxidation [45]. Moreover, high concentration polyphenol promotes the bridging of protein chains to form larger metastable colloids. This indicates that due to the interaction between casein and polyphenols, the thickness of the oil-water interface gradually increases, effectively slowing down the oxidation of linoleic acid [45]. Recently, the interaction of polyphenols with proteins has attracted much attention in the field of food nutrition. These casein-polyphenol complexes may alter the nutritional potential of polyphenols by affecting their anti-inflammatory and antioxidant capacity. On the other hand, they may affect the structure of proteins, thereby altering the physicochemical properties of the system, such as protein solubility, bioaccessibility, emulsification, and foamability [46]. For example, it has been reported that the combined use of polyphenols with casein could effectively improve the stability and emulsification effect of dairy products [47]. In addition, the best process conditions should also be explored in detail to improve food quality and provide the most beneficial health effects for consumers with the best nutritional characteristics. This might help develop functional foods that contain both polyphenols and casein. In this section, the interaction between polyphenols and caseins is introduced in detail.

### 3.1. Types of Polyphenols

**Figure 2:**
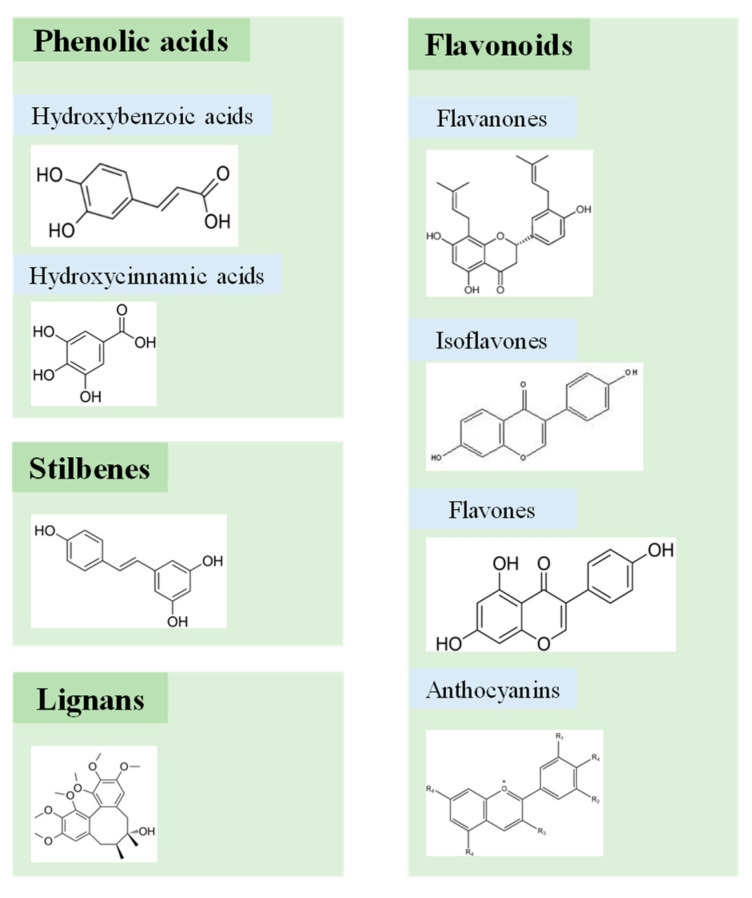
Main categories and chemical structures of fruit and vegetable phenolics.

Polyphenols are a general term for a class of plant secondary metabolites with phenol as the basic skeleton and polyhydroxyl substitution of the benzene ring as the main feature. Up to now, more than 8000 structures have been discovered for polyphenols [42]. According to the number and location of aromatic rings and associated hydroxyl groups, polyphenols are mainly categorized into phenolic acids, flavonoids, stilbenes, and lignans (Figure 2) [48]. Phenolic acids are organic acids containing phenolic rings and at least one carboxylic group, which consist of two subset clarifications (i.e., hydroxybenzoic acids and hydroxycinnamic acids). Flavonoids are a class of compound based on the 2-phenylchromogenone-4-ketone skeleton, including flavanones, isoflavones, flavones, and anthocyanins, as differentiated by the connection position of the C-ring and B-ring and the degree of unsaturated C-ring [48,49]. In comparison, stilbenes (C_6_-C_2_-C_6_) and lignans ((C_6_-C_3_)n) are relatively less prevalent polyphenols [50].

### 3.2. Interaction Types

In the past few decades, the interaction between polyphenols and some plant-sourced proteins has been widely studied. It is well recognized that polyphenol-protein interactions mainly occur through non-covalent and covalent bonds [42,51], the occurrence of which can significantly affect the structural and functional properties of the proteins, such as enzyme activity, protein digestibility, and antioxidant properties [52].

The interaction between polyphenols and casein has also been well studied. Based on the results of fluorescence analysis, the binding constants of casein-tannic/gallic acid complex were reported to be in the range of 10^4^ to 10^7^ L mol^−1^. In addition, scanning electron microscopy also showed that the addition of polyphenols led to a looser casein structure [53]. Notably, the binding of polyphenols and casein is generally affected by several factors, such as temperature, pH, and the structure of polyphenols [46]. In this section, the interaction types and influencing factors between casein and polyphenols are introduced.

#### 3.2.1. Non-Covalent Interactions

**Figure 3:**
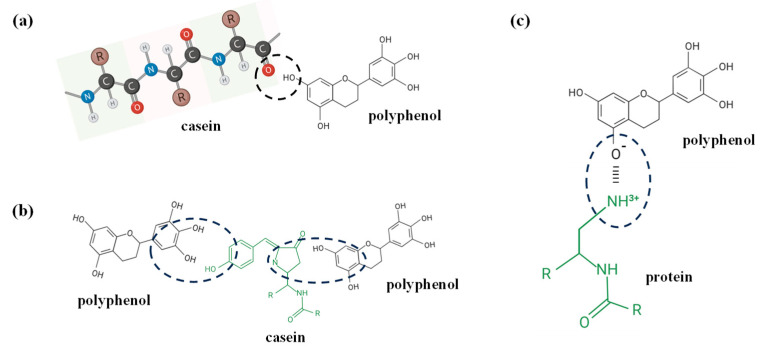
Non-covalent conjugation of casein and polyphenol and protein cross-linking via (**a**) hydrogen bonding, (**b**) hydrophobic-hydrophobic interaction, and (**c**) ionic interaction adopted from Quan et al. [51].

Polyphenols and casein can form conjugates through non-covalent interactions, including hydrogen bonding, hydrophobic interaction, ionic bonding, and van der Waals force (Figure 3) [42,54,55]. Hydrogen bonding (Figure 3a) is one of the main forces involved in the formation process of casein-polyphenol conjugates, with the C=O of proteins readily forming hydrogen bonds with phenolic groups. Moreover, the binding sites of hydrogen bonds are mainly glutamic acid and glutamine residues [47]. The interaction between the hydroxyl groups of polyphenols and oxygen or nitrogen in casein can also form hydrogen bonds [51], which can enhance the solubility of polyphenols and confer better encapsulation and delivery capacities on casein-polyphenol nanoparticles [56]. Hydrophobic interactions can be formed between hydrophobic amino acids in casein (e.g., phenylalanine, isoleucine, tyrosine, alanine) and nonpolar aromatic rings of polyphenols (Figure 3b) [57]. The occurrence of hydrophobic interactions may result in structural changes in casein, which usually occur when a benzene ring present in a polyphenol compound binds to a hydrophobic group such as an alkyl group in the protein [58]. In addition, ionic bonds are another common form of non-covalent interaction, which can be formed by positively charged protein groups (e.g., ε-amino of lysine) reacting with negatively charged polyphenol hydroxyl groups (Figure 3c) [59]. Additionally, the presence of van der Waals forces further enhances the polyphenol-protein binding ability [60]. Usually, there are two or more non-covalent forces in casein-polyphenols complex systems [51]. Hydrogen bonds and hydrophobic interactions are generally the main forces governing non-covalent binding between polyphenols and proteins, whereas ionic bonds play a secondary role. Chanphai et al. [61] reported that the casein residues of Phe 23, Phe 28, Phe 32, and Val 31 bind to catechins through hydrogen bonding and hydrophobic interactions. Moreover, it has been demonstrated that gallic acid, epigallocatechin gallate, quercetin, and quercetin can interact with α-casein and β-casein and form complexes mainly through hydrogen bonding, hydrophobic interactions, and van der Waals forces [62]. These phenolic compounds can approach the hydrophobic cavities of α-casein and β-casein, and the nonpolar aromatic rings of phenolic compounds can interact with the residues of hydrophobic amino acids such as proline and valine through hydrophobic interactions.

#### 3.2.2. Covalent Interactions

**Figure 4:**
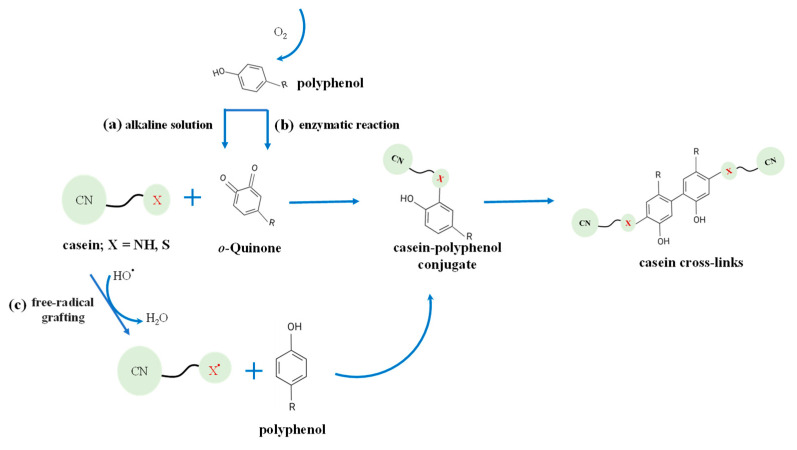
Covalent conjugation of casein and polyphenol and protein cross-links via (**a**) alkaline, (**b**) enzymatic, and (**c**) free-radical grafting reactions adopted from Quan et al. [51].

Covalent interactions are generally irreversible and stronger than their noncovalent counterparts [63]. For a mixture of polyphenol and casein, covalent interactions usually occur under alkaline and oxygen conditions, mainly through the oxidation and nucleophilic addition of polyphenols [51,62,64]. Generally, polyphenols are easily oxidized in the presence of oxygen under alkaline conditions (pH 9.0) to generate semiquinone radicals and rearrange into quinones. These reactive intermediates readily interact with nucleophilic residues (such as methionine and tryptophan) in the protein side chain to produce covalent crosslinks (C-N or C-S) (Figure 4a) [65]. An example is that the covalent binding of quercetin and casein was achieved under alkali treatment (pH 9.0), which conferred improved solubility to casein [66]. The tryptophan (Trp) residue of casein can be oxidized to quinone, which further forms covalent conjugate with quercetin through a nucleophilic reaction with polyphenols. Similar results have also been reported for covalent complexes of whey protein isolate and quercetin prepared by free grafting [63].

Enzyme-catalysis is a highly specific method but involves a complicated procedure (Figure 4b). The first step is to induce the oxidation of monophenolase or cresolase to produce *o*-diphenols. Second, the *o*-diphenol enzyme or catecholate acts to convert *o*-diphenols into *o*-quinones in the presence of oxygen. A typical example of the catalytical synthesis of protein-polyphenols conjugates is through laccase. Laccase is a polyphenol oxidase with high thermal stability and strong oxidation ability, and laccase catalysis is usually considered as an effective method to promote protein-polyphenol covalent interactions. It can oxidize *o*-diphenols and *p*-diphenols to produce quinones, which readily bind to nucleophilic amino acid residues in protein chains [67]. A previous study showed that laccase could facilitate the binding of gallic acid to soy protein isolate (SPI) at neutral pH, resulting in a nearly five-fold increase in DPPH radical clearance compared with unmodified SPI [68]. However, few reports are available at present regarding the use of laccase to catalyze the covalent interaction between casein and polyphenols, which may be an interesting topic in the future.

Free radical grafting, a non-enzymatic method, is also considered a useful method to synthesize protein-polyphenol conjugates (Figure 4c). Generally, hydroxyl radicals produced from the redox pair reaction of hydrogen peroxide and ascorbic acid can oxidize amino acids located on the protein side chains. Subsequently, the free radicals located on the proteins react with the phenol ring of the polyphenol to form covalent bonds. Furthermore, heteroatom-centered radicals on protein side chains tend to react at *ortho-* and *para-* sites compared with the hydroxyl group on the polyphenol ring [69]. Gu et al. [70] previously demonstrated that catechin-egg white protein conjugates synthesized from free radical grafting possessed higher DPPH radical scavenging capacity because of the presence of the phenolic hydroxyl groups of catechin in the conjugated backbone. Similarly, free radical grafting of casein by caffeic acid also increased the emulsifying activity and stability of casein by 1.65 times and 1.38 times, respectively [71].

### 3.3. Influencing Factors

The factors affecting the interaction between casein and polyphenols mainly include pH, temperature, the mixing ratio of casein/polyphenol, and the structural characteristics of polyphenols.

#### 3.3.1. pH

pH is a crucial factor affecting the formation of casein-polyphenol complexes. At pH < 7, casein undergoes dissociation, resulting in the exposure of binding sites that can interact with polyphenols through electrostatic interactions. However, at pH > 7, polyphenols are easily oxidized to produce active free radicals and quinones and thus lead to the occurrence of covalent interactions between casein and polyphenols through non-enzymatic oxidation, as mentioned above. [57]. This suggests that pH is an important factor affecting the casein conformation or polyphenol structure. By adjusting the pH, the binding mode of polyphenol and casein can be modulated, which can further change the functional properties of casein. Previously, Ke et al. [66] reported that the covalent binding of quercetin with casein in an alkaline environment can improve the solubility and emulsification activity of casein compared with the non-covalent approach.

#### 3.3.2. Temperature

Temperature is another major factor affecting casein-polyphenol interaction. Increasing temperature usually causes conformational changes in proteins and leads to exposure of anteriorly buried hydrophobic points, thus favoring the binding of proteins to polyphenols through hydrophobic interactions [51,72]. It was previously reported that raising temperature increased the number of binding sites of β-casein in donkey and bovine milk, and the reason was attributed to the change in the surface hydrophobicity of β-casein as affected by heating [73]. In addition, under heating, polyphenols are easily oxidized to form quinone derivatives, which may also initiate the covalent binding of polyphenols to proteins [74].

#### 3.3.3. Mixing Ratio of Protein/Polyphenol

**Figure 5:**
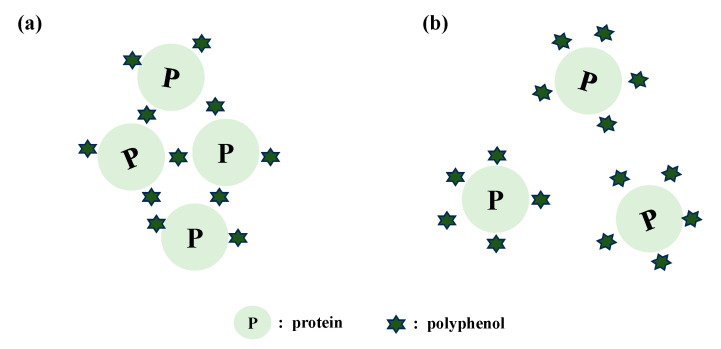
The (**a**) multidentate and (**b**) monodentate mechanism of protein-polyphenol interaction adopted from Günal-Köroğlu et al. [42].

The mixing ratio can affect the protein-polyphenol interaction through two mechanisms: multidentate and monodentate mechanisms (Figure 5) [75]. At a high protein/polyphenol ratio (meaning that polyphenol is unsaturated relative to protein), the interaction sites of proteins and polyphenols (multi-site ligands) can directly produce intermolecular bridges (cross-links between proteins) (Figure 5a). Conversely, at a high polyphenol concentration, the interaction site of the protein chain is saturated, leading to the binding of various polyphenol substances to one protein (Figure 5b). In this case, polyphenol/protein conjugates can be formed more easily. However, excessive addition of polyphenol may lead to competitive aggregation between polyphenol/protein conjugated and protein self-aggregation, which may reduce the possibility and strength of interaction between polyphenol and protein molecules. Xue et al. [76] reported that the binding content of ferulic acid was the highest when the mass ratio of β-lg to ferulic acid was 10:6. Moreover, Pascal et al. [77] found that when the concentration of human salivary proline-rich protein (IB-5) was low, increasing the concentration of epigallocatechin gallate enhanced its binding ability to proteins, whereas excessive addition of epigallocatechin gallate induced the formation of IB-5 aggregates. When the IB-5 concentration is sufficiently high, a small amount of epigallocatechin gallate addition can cause remarkable aggregation of the protein.

#### 3.3.4. Structure of Polyphenols

Different structures of polyphenols can also affect their binding to casein. Usually, the molecular parameters of polyphenols such as molecular weight, hydroxylation, and glycosylation can significantly affect casein-polyphenol interactions [78]. Chen et al. [47] reported that polyphenols with higher molecular weight and more phenolic hydroxyl groups possessed a higher binding sensitivity to casein. For example, gallic acid, epigallocatechin, and epigallocatechin gallate have higher affinity for casein binding. Meanwhile, Chanphai et al. [61] also found that larger phenolic compounds are more likely to form a stable system with β-lg, and their binding strength was in the order of epigallocatechin gallate > epicatechin gallate > epicatechin > catechin. In addition, glycosylation facilitates the formation of more stable polyphenol/casein complexes. It was found that the binding affinity of quercitrin (4.8383 × 10^5^~3.4522 × 10^6^ L/mol) to α-casein was much larger than that of quercetin (2.6019 × 10^3^~1.4631 × 10^4^ L/mol) [47].

Similar to proteases, polyphenols can stabilize or disrupt the structure of protein substrates after binding to proteins, making them less or more susceptible to protein hydrolysis attacks. However, the bioavailability of proteins depends on the degree of structural stability or instability of enzymes and protein substrates, as well as the extent to which enzymes and protein substrates are bound non-covalently, or covalently shielded by polyphenols. Therefore, the results of the bioavailability of polyphenol-casein complexes vary under different conditions, including polyphenol/protein ratio, enzyme/protein ratio, digestion time, and different evaluation methods [67]. For example, after non-covalent binding with catechins, the α—helix and β—sheet in casein decreased, reducing its stability [79]. On the other hand, when tea polyphenols are bound to milk proteins (α- and β-casein), they spatially hinder the approach of gastric proteases, thereby increasing the stability of casein during digestion [80].

## 4. Interactions with Metal Ions

Metal ions play an important role in the process of biological life, not only participating in gene expression and enzymatic catalysis, but also modulating some important physiological activities such as body growth, metabolism, and so on [81]. Metal ions are especially important to the synthesis and functionalities of proteins because about 30% of proteins are metalloproteins.

Casein is composed of four gene products, α_s1_-, α_s2_-, β-, and κ-casein, varying in structure and degree of post-translational modification [8]. Phosphorylated serine residues of casein are able to bind to calcium ions and associate to form globular aggregates (casein micelles). The process of casein separation is usually accompanied by the distribution of milk minerals (especially calcium and zinc) between casein micelles and serum components. Therefore, casein can be used as a natural carrier of metal ions [8,82,83]. Recently, metal-casein has attracted great attention, mainly because metal-based complexes have been demonstrated to show great potential for applications in food processing, pharmacology, or medicine [84].

### 4.1. Calcium

Calcium has important physiological functions in controlling metabolism and hormone secretion. For milk casein, the content of minerals (especially calcium and magnesium) can affect the distribution and aggregation of casein considerably [85]. The binding of metal ions to casein is believed to be through negatively charged aspartate and glutamate carboxylic groups and phosphoserine residues in casein [12].

Casein monomer components contain multiple phosphoserine residues of phosphate groups, which can effectively bind to calcium ions, reduce the effect of electrostatic stability mechanism, and lead to the association of caseinate submicelles [86]. According to the difference of phosphate content in the casein monomer, the sensitivity of casein to calcium ions follows the order of α_s_-casein > β-casein > κ-casein [12]. In addition, calcium can bind to the phosphoric acid group of α_s_-casein and the N-terminal carboxyl group of β-casein, both of which have a central SerP-SerP-SerPX-SerP sequence that forms the central position of casein, allowing it to bind numerous calcium ions [4]. Nonetheless, κ-casein contains only one phosphoserine residue, and this special chemical structure leads to its strong hydrophilicity. In the presence of Ca^2+^, κ-casein can inhibit the precipitation of α_s_-casein and β-casein, thus stabilizing the spatial structure of casein micelles [12,87].

### 4.2. Silver

Due to the strong antibacterial effect of silver ions and the great biological activities (e.g., immunomodulatory, antioxidant, and anticancer) of silver nanoparticles, the application of silver ions has received much attention [12,88,89]. Based on Pearson’s Hard and Soft (Lewis) Acid-base (HSAB) principle, Ag^+^ ions are “soft” cations and are more likely to interact with soft ligands [88]. This indicates that silver ions are more likely to interact with methionine or cysteine side chains because the thio-containing group is considered to be the softest ligand [12].

Compared with other globular proteins, casein has a more complex structure, which may affect its binding capacity to silver ions [12]. Generally, the interaction of Ag^+^ ions with casein is a multiphase process with two main stages [12,88]. First, Ag^+^ ions are rapidly absorbed to the surface of casein; then, Ag^+^ ions diffuse at a slower rate and bind within the lobules. It is worth noting that FT-IR spectroscopy recently confirmed that silver ions can also bind to the carboxyl group of casein’s aspartic acid and glutamate [12]. The results of surface enhanced Raman spectroscopy results also indicate the critical role of carboxylic groups in stabilizing aspartate and glutamate residues as well as the role of phosphate groups in immobilizing silver ions onto bovine lactoferrin [90].

### 4.3. Zinc

Zinc is an essential trace element in the human body, participating in regulation of the functional properties of macromolecules and enzymes [91]. Due to the inability of the human body to store zinc, daily dietary intake of zinc is thus required for maintaining health [84,89].

However, the addition of zinc may affect the stability of milk casein. It has been demonstrated that Zn^2+^ can bind to proteins through a few amino acid residues, including aspartate, glutamic acid, cysteine, histidine, and aspartate, as well as phosphoserine [84]. For casein, Pomastowski, Sprynskyy, and Buszewski [92] and Rodzik et al. [84] confirmed that the binding process of Zn^2+^ to casein was through casein’s Asp- and Glu- carboxyl groups. They also found that Zn^2+^ ions may bind to phosphate groups of serine, imidazole rings of histidine (mainly nitrogen), or deprotonated carboxyl groups of glutamate and aspartic acids. Additionally, in some cases, Zn^2+^ may be involved in the cross-linking of two or more adjacent charged amino acid groups [84,92].

### 4.4. Other Considerations

Metal ions facilitate the release of casein-derived titanium, and they are beneficial to the body’s regulatory functions, such as anti-inflammatory and antihypertensive activities [92]. Casein-metal ions complexes have potential applications as antimicrobial agents (e.g., Gram-positive bacteria) and dietary supplements, which is of considerable significance for preparation of casein-metal ions conjugates for microelement deficiency group [12,90,91,92]. Factors such as changes in casein conformation and possible casein aggregation are critical for assessing changes in casein functional properties caused by interactions with metal ions [91]. The Derjagin–Landau–Verwey–Overbeek (DLVO) theory elucidates that the balance between the attractive van der Waals force and the repulsive double-layer electricity can affect the stability of biocolloidal systems [93]. Therefore, the addition of metal ions increases the attraction, thus strengthening the interaction between the protein units, resulting in the formation of large aggregates [91]. For example, the synergistic action of iron, chromium, and nickel ions causes the aggregation of human serum albumin (HSA) protein [94]. Based on the above studies, we speculate that metal ions (such as iron, magnesium, and calcium ions) may also cause casein aggregation. Metal-protein interactions have been applied to produce nanoparticles, metallocomplexes, and metalloproteins. It has been reported that zinc successfully binds to milk proteins to produce metallocomplexes such as zinc-casein [92,95] and zinc-β-lg, as well as nanocomposites such as zinc-oxy-ovalbumin [96]. Furthermore, properties of metal ions such as valence, ionic radius, free metal concentration, and charge-accepting capacity also affect the interaction of metal ions with casein [89,91]. However, the studies of functional properties of metal-casein interactions and changes in characterization of metal-casein binding are still challenging and need to be explored through a combination of instrumental techniques and data analysis techniques.

## 5. Conclusions and Future Prospects

Casein is an important protein in milk, serving structural and nutritional functions. Polysaccharides, polyphenols, and metal ions are widely found in plants and animals, and they are often added to dairy products as important ingredients to improve the functional properties and quality of dairy products. In the case of polysaccharides, thermodynamic incompatibility and attraction are the fundamental factors determining the interaction types of casein-polysaccharide. Casein-polyphenol interactions occur primarily through non-covalent (hydrogen bonding, hydrophobic interactions, ionic bonding, and van der Waals forces) and covalent (enzymatic or non-enzymatic oxidation) binding. The specific selectivity of casein to metal ions facilitates their combination to form metal proteins, nanoparticles, and metal complexes. However, studies of the functional properties of metal-protein interactions and changes in the characterization of metal-protein binding are still challenging. In addition, factors such as temperature, pH, the mixing ratio, as well as the fine structure of these components significantly affect the binding of casein to the three components. Therefore, in order to develop dairy products with higher nutritional value, a deeper understanding of the binding of casein to different components in food is needed. In addition, before conducting in-depth research, it is necessary to fully understand and master the instrumental techniques and data analysis techniques.

## Data Availability

The original contributions presented in the study are included in the article, further inquiries can be directed to the corresponding authors.
